# Post-myocardial Infarction Ventricular Septal Defect in the Setting of No-Reflow and COVID

**DOI:** 10.7759/cureus.41525

**Published:** 2023-07-07

**Authors:** Abdul Haseeb Riaz, Usman Younus

**Affiliations:** 1 Internal Medicine, Campbell University, Cape Fear Valley Medical Center, Fayetteville, USA; 2 Critical Care, Cape Fear Valley Medical Center, Fayetteville, USA

**Keywords:** no-reflow, post mi ventricular septum rupture, ventricular septal defect (vsd), vsd, covid

## Abstract

The occurrence of post-myocardial infarction (MI) ventricular septal defect (VSD) is a rare but life-threatening complication. This case report presents a unique case of a 49-year-old female patient with an anterolateral ST-segment elevation MI who underwent percutaneous coronary intervention (PCI) and drug-eluting stent (DES) placement, complicated by a no-reflow phenomenon in the distal left anterior descending artery (LAD) and subsequent development of a hemodynamically significant VSD. Notably, this case occurred during the COVID-19 pandemic, which added to the complexity of the patient's management. The patient's clinical course was further complicated by cardiogenic shock, acute respiratory failure, COVID-19 pneumonia, and gastrointestinal bleeding. Despite these challenges, the patient received prompt treatment and optimal medical management, including the use of vasopressor support, insulin therapy, and bicarbonate infusions. The patient also underwent surgical repair of the VSD at a quaternary center, resulting in a favorable outcome. This case report highlights the increased incidence of mechanical complications, such as VSD, during the COVID-19 pandemic due to delayed presentation and patient concerns about exposure to the virus. It also emphasizes the occurrence of a no-reflow phenomenon during PCI, which can lead to adverse outcomes, including larger infarct size and potential ventricular septal rupture. The case further underscores the importance of multidisciplinary collaboration and early subspecialist involvement in managing complex cases of post-MI VSD.

## Introduction

Ventricular septal defects (VSDs) are among the most common congenital heart disease; however, they can also occur due to acquired causes such as aortic valve replacement, Takotsubo cardiomyopathy, and septal myomectomy for hypertrophic obstructive cardiomyopathy (HOCM) [1]. The most common acquired cause is an acute myocardial infarction (MI) [2]. Although the post-MI VSD incidence has been declining due to advances in ST-elevation myocardial infarction (STEMI) care and early reperfusion, an increasing number of cases were reported in the literature during the COVID-19 pandemic [3].

Post-MI VSDs usually occur in a bimodal distribution, i.e., within the first 24 hours after MI and three to five days after MI. They are caused by disruption of blood flow to the interventricular septum resulting in ischemic necrosis and transmural infarct, which cause a rupture between the necrotic and non-necrotic myocardium. VSDs occur in the anterior or apical portion of the ventricular septum in cases of anterior MI and in the posterior portion as a result of posterior MI. The clinical manifestations include chest pain, shortness of breath, hypotension, acute heart failure, and cardiogenic shock [4,5]. A new loud holo-systolic murmur is characteristic, and a thrill can be palpated in up to 50% of the patients [6]. Diagnosis is confirmed with echocardiography [7]. Its incidence has decreased to <0.3% due to early reperfusion strategies [8-10]. They are associated with extremely high mortality with an estimated survival rate of 8% at 30 days and <3% at one year [11]. Risk factors include age, gender, single vessel disease, especially occlusion of the left anterior descending artery (LAD), poor septal collaterals, and late presentation [4,12].

In this article, we discuss a case report of a patient with COVID-related post-MI VSD, which was complicated by the no-reflow phenomenon after percutaneous coronary intervention (PCI).

## Case presentation

**Figure 1:**
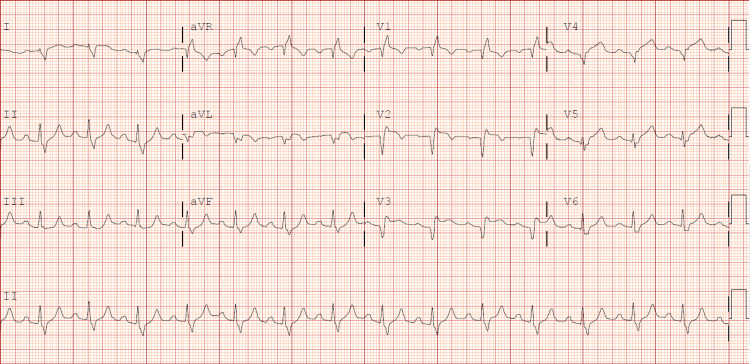
EKG showing ST-segment elevations in leads I, aVL, V1-V5 along with reciprocal ST-segment depressions in II, III, and aVF EKG: Electrocardiogram; aVL: Augmented vector left; aVF: Augmented vector foot.

A 49-year-old female with a past medical history of tobacco use, diabetes, schizophrenia, anxiety, and depression presented with left-sided, non-radiating chest pain along with nausea, diaphoresis, and shortness of breath, which started about 18 hours ago. Her presentation was delayed due to hesitancy for hospitalization due to the fear of COVID-19 exposure. She was hypotensive on arrival with initial lab work significant for hyponatremia with sodium of 129, hyperkalemia with potassium of 6.0 acute kidney injury with creatinine of 1.51, hyperglycemia with glucose of 593, leukocytosis with WBCs of 20.8. An electrocardiogram (EKG) revealed ST elevations in leads I, II, aVL, and V1-V5 (Figure 1) with high-sensitivity troponin greater than 25,000.

**Figure 2:**
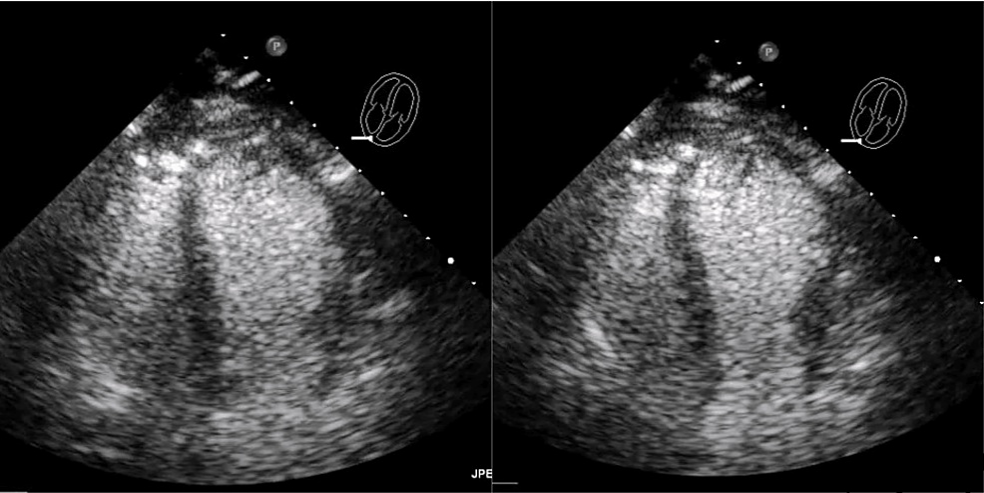
Transthoracic echocardiography in an apical four-chamber view shows contrast enhancement in systole and diastole, showing visually reduced EF and an intact interventricular septum EF: Ejection fraction.

An initial echocardiogram on arrival revealed wall motion abnormality consistent with an anteroapical infarction and reduced left ventricle (LV) systolic function with an ejection fraction of 30%-35%. The interventricular septum was intact without any signs of weakening (Figure 2).

Aspirin and heparin were administered, and the patient was taken urgently for a left heart catheterization, which revealed 99% thrombotic occlusion of proximal LAD, with the right coronary artery (RCA) and left circumflex artery (LCX) noted to have mild luminal irregularities. Balloon angioplasty was performed, and a drug-eluting stent (DES) was placed in the proximal LAD; however, after PCI was noted to have no reflow in distal LAD likely due to the longer period of coronary occlusion and reperfusion injury. Due to hypotension requiring vasopressors, an intra-aortic balloon pump (IABP) was placed with 1:1 augmentation.

**Figure 3:**
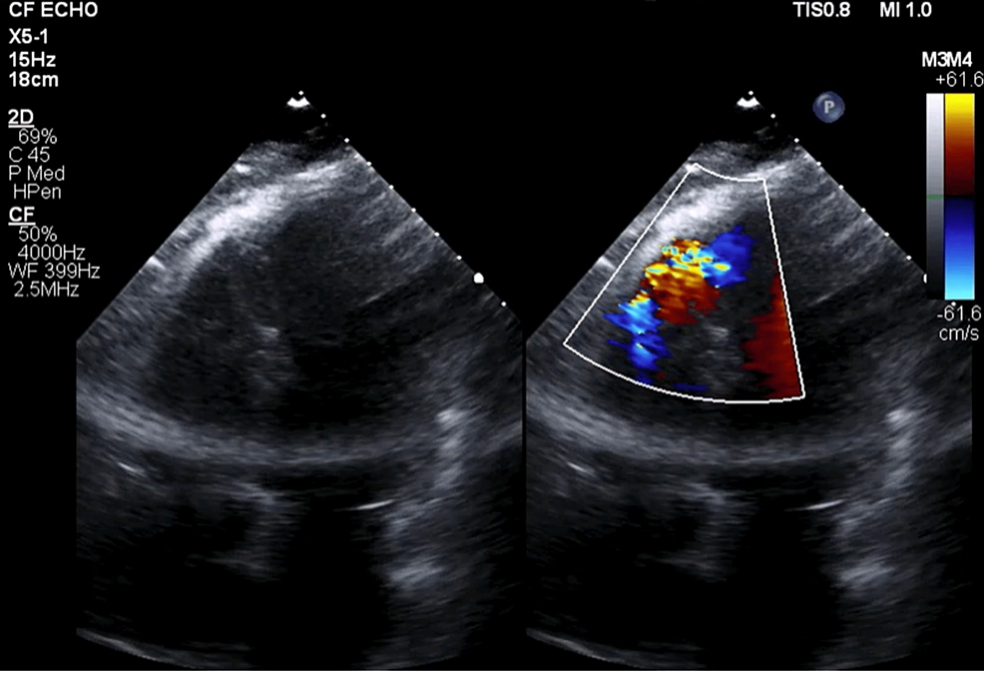
TTE in an apical four-chamber view, showing VSD with left-to-right shunt on Doppler imaging TTE: Transthoracic echocardiogram; VSD: Ventricular septal defect.

The patient was then transferred to ICU for post-PCI care. She was placed on vasopressor support and was treated for diabetic ketoacidosis (DKA) with insulin and bicarbonate infusions. The ICU course was complicated by cardiogenic shock, acute hypoxemic respiratory failure from acute pulmonary edema, COVID-19 pneumonia, and GI bleeding. Infectious disease recommended treatment of COVID-19 with dexamethasone and tocilizumab. Her pressor requirement was weaned on day 3, and the IABP was removed. She was started on low-dose metoprolol but unfortunately became hypotensive again on day 4 requiring the IABP to be reinserted and pressors to be restarted. A repeat echocardiogram at this juncture was obtained, which identified a muscular VSD at least 1 cm in diameter with a prominent left-to-right shunt along with severely reduced LV function (Figure 3).

Given the complexity of the procedure, the patient was transferred to a quaternary center where she underwent successful surgical VSD repair 11 days after the discovery of VSD. A two-patch technique with bovine pericardium patches was used for VSD closure. The patient had a gradual recovery and was discharged home. Prompt treatment and optimum medical management resulted in favorable outcomes.

## Discussion

During the COVID-19 pandemic, hospitals witnessed a reduction in admissions and an increased time to admission, which resulted in a larger number of patients who were outside the time window for PCI. This is theorized to be due to the patients’ fear of being exposed to COVID-19. As a result, it was noted that the incidence of mechanical complications, such as VSD, increased [12].

Coronary no-reflow is a frequent but challenging occurrence during PCI. It is more common in late presentation anterior MI. The primary cause of no-reflow is microvascular blockage, which can be brought on by microvascular spasm, intravascular plugging from platelet micro-thrombi or leukocytes, distant embolization of thrombus or debris, or ischemia-reperfusion injury. Female sex, age > 65 years, hypertension, diabetes, hyperlipidemia, CHADS_2_-VASc (congestive heart failure, hypertension, age ≥75, diabetes, stroke (doubled), vascular disease, age 65 to 74 and sex category [female]) score ≥ 3.5, delayed presentation for STEMI patients, and renal insufficiency place patients at a higher risk. No-reflow results in an increased incidence of systolic heart failure, malignant arrhythmias, and larger infarct size, which may predispose patients to ventricular septal rupture. Liberal vasodilator use and thrombectomy are recommended treatments for the no-reflow phenomenon [13,14].

The best time to repair a post-MI VSD is still contested; however, early repair (usually defined as within one week of presentation) is linked with a mortality rate of 20%-40% and a high risk of recurrent ventricular rupture. On the other hand, late repair while beneficial in terms of the organization of infarcted tissue carries the danger of rupture extension and death while awaiting operational intervention. Long-term closure delays may potentially contribute to the progression of pulmonary hypertension and right heart failure [15]. The American Heart Association (AHA) recommends immediate afterload reduction as a cornerstone of initial therapy, and peri-procedural temporary mechanical support as a helpful addition to decompress the LV and promote cardiac output. Emergent repair is suggested if hemodynamic instability, especially cardiogenic shock or respiratory failure, is refractory to mechanical circulatory support. If the patient is hemodynamically stable, the AHA suggests delaying surgery and optimal timing to be discussed with a multi-specialty team of cardiac surgeons, cardiologists, and intensivists. Patients who are not candidates for VSD surgery have options such as percutaneous closure, mechanical support for heart transplants, and palliative medical therapy [10]. Since our patient's hemodynamics remained relatively stable and vasopressor requirements were not increasing, the multidisciplinary team decided on a delayed repair approach, and surgery was performed 11 days after the discovery of VSD.

This case report adds to the existing literature on post-MI VSD by highlighting the potential increased incidence of mechanical complications during the COVID-19 pandemic due to delayed presentation and fear of exposure to the virus. It highlights the occurrence of a no-reflow phenomenon during PCI as a result of the increased period of coronary occlusion due to delayed presentation and the subsequent development of VSD. The presence of active COVID-19 infection likely contributed to no-reflow as well because prior literature has shown elevated D-dimer to be an independent predictor of no-reflow phenomenon, and patients with COVID-19 infection, like our patient, frequently have elevated D-dimer [16]. This case report furthermore contributes to the existing literature by providing a real-life example of the challenges and management strategies for post-MI VSD in the context of the COVID-19 pandemic.

## Conclusions

After all MIs, it is important to be aware of the uncommon and potentially fatal complication known as post-MI VSD. This has also been influenced by the COVID-19 pandemic. It is necessary to explore more aggressive treatment for the no-reflow phenomenon. Hemodynamic support and early subspecialist involvement should be implemented to enhance results.
